# Allergen-specific immunotherapy

**DOI:** 10.1186/1710-1492-7-S1-S5

**Published:** 2011-11-10

**Authors:** William Moote, Harold Kim

**Affiliations:** 1 Division of Clinical Immunology & Allergy, University of Western Ontario, London, Ontario, Canada; 2 University of Western Ontario, London, Ontario, Canada; 3 McMaster University, Hamilton, Ontario, Canada

## Abstract

Allergen-specific immunotherapy is a potentially disease-modifying therapy that is effective for the treatment of allergic rhinitis/conjunctivitis, allergic asthma and stinging insect hypersensitivity. However, despite its proven efficacy in these conditions, it is frequently underutilized in Canada. The decision to proceed with allergen-specific immunotherapy should be made on a case-by-case basis, taking into account individual patient factors such as the degree to which symptoms can be reduced by avoidance measures and pharmacological therapy, the amount and type of medication required to control symptoms, the adverse effects of pharmacological treatment, and patient preferences. Since this form of therapy carries the risk of anaphylactic reactions, it should only be prescribed by physicians who are adequately trained in the treatment of allergy. Furthermore, injections must be given under medical supervision in clinics that are equipped to manage anaphylaxis. In this article, the authors review the indications and contraindications, patient selection criteria, and the administration, safety and efficacy of allergen-specific immunotherapy.

## Introduction

Allergen-specific immunotherapy (also known as allergy shots) is an effective treatment used by allergists and immunologists for common allergic conditions, particularly allergic rhinitis/conjunctivitis, allergic asthma and stinging insect hypersensitivity [1-4]. This form of therapy typically involves the subcutaneous administration of gradually increasing quantities of the patient’s relevant allergens until a dose is reached that is effective in inducing immunologic tolerance to the allergens. The primary objectives of allergen-specific immunotherapy are to decrease the symptoms triggered by allergens and to prevent recurrence of the disease in the long-term. Currently, it is the only identified disease-modifying intervention for allergic disease [5,6].

Despite the proven efficacy of immunotherapy for the treatment of allergic conditions, it is frequently underutilized in Canada [6]. This article will review the mechanisms of immunotherapy, its indications and contraindications, patient selection criteria, and the administration, safety and efficacy of this form of therapy.

## Mechanisms of immunotherapy

Immunologic changes that occur during allergen-specific immunotherapy are complex and not completely understood. However, successful immunotherapy has been associated with a shift from T helper cell type-2 (Th2) immune responses, which are associated with the development of atopic conditions, to Th1 immune responses. It is also associated with the production of T regulatory cells that produce the anti-inflammatory cytokine, interleukin 10 (IL-10), amongst others such as transforming growth factor (TGF)-beta. IL-10 has been shown to reduce levels of allergen-specific immunoglobulin E (IgE) antibodies, increase levels of immunoglobulin G (IgG) (blocking) antibodies that play a role in secondary immune responses, and reduce the release of pro-inflammatory cytokines from mast cells, eosinophils and T cells. Allergen-specific immunotherapy has also been found to decrease the recruitment of mast cells, basophils, and eosinophils to the skin, nose, eye, and bronchial mucosa after exposure to allergens, and reduce the release of mediators, such as histamine, from basophils and mast cells [5,7]. Research surrounding the mechanisms of immunotherapy is still ongoing and will help further elucidate how this form of therapy exerts its beneficial effects in allergic diseases.

## Indications

Allergen-specific immunotherapy is indicated in patients with allergic rhinitis/conjunctivitis and/or allergic asthma who have evidence of specific IgE antibodies to clinically relevant allergens (see Table 1). Skin prick testing (SPT) is the preferred method of testing for specific IgE antibodies. Allergen-specific IgE testing which provides an *in vitro* measure of a patient’s specific IgE levels against particular allergens is a reasonable alternative to SPT. However, SPTs are generally considered to be more sensitive and cost effective than allergen-specific IgE tests [5-7]. Patients with allergic rhinitis/conjunctivitis or allergic asthma who may be good candidates for immunotherapy include those who: have symptoms that are not well controlled by pharmacological therapy or avoidance measures; require high doses of medication, multiple medications, or both to maintain control of their disease; experience adverse effects of medications; or wish to avoid the long-term use of pharmacological therapy [7].

**Table 1:** Allergen-specific Immunotherapy: indications, contraindications and special considerations [5-7]

Indications:	• Patients with stinging insect (venom) hypersensitivity • Patients with allergic rhinitis/conjunctivitis and/or allergic asthma who have evidence of specific IgE antibodies to clinically relevant allergens; includes patients who: – Do not achieve control of symptoms with avoidance measures and pharmacotherapy – Do not want ongoing or long-term pharmacotherapy – Experience undesirable side effects with pharmacotherapy
**Contraindications:**	• Patients on beta-blockers (relative contraindication with venoms) • Patients with uncontrolled or severe asthma • Significant co-morbid diseases such as cardiovascular disability

**Special considerations:**	• Children < 6 years of age • Pregnancy • The elderly • Patients with malignancy, immunodeficiency and autoimmune diseases

IgE: immunoglobulin E

Venom immunotherapy is indicated in individuals of all ages with severe systemic reactions to stinging insects, as well as in adults who experience generalized reactions that are limited to the skin (see Table 1) [6]. Severe systemic reactions to Hymenoptera (classification of insects that includes bees and wasps) venom are relatively uncommon, but can be fatal. The purpose of venom immunotherapy is to reduce the severity of the reactions and the risk of fatality, and to improve patient quality of life by allowing the patient to work or play outdoors without being concerned about the possibility of experiencing a serious allergic reaction [5].

## Contraindications

Allergen-specific immunotherapy is contraindicated in patients with medical conditions that increase the patient’s risk of dying from treatment-related systemic reactions, such as those with severe or poorly controlled asthma or significant cardiovascular diseases (e.g., unstable angina, recent myocardial infarction, significant arrhythmia, and uncontrolled hypertension) (see Table 1). Immunotherapy is also contraindicated in patients using beta-blockers since these agents can amplify the severity of the reaction and make the treatment of systemic reactions more difficult. Immunotherapy should be considered, however, in patients with life-threatening stinging insect hypersensitivity, even if they also require beta-blocker medications, because the fatal risk associated with an insect sting is greater than the risk of an immunotherapy-related systemic reaction [6,7].

## Special considerations

Special consideration should be given to the use of allergen-specific immunotherapy in children under 6 years of age, pregnant women, the elderly, and patients with malignancy, or immunodeficiency/autoimmune diseases (see Table 1). Immunotherapy is effective in children and is often well tolerated. However, children less than 6 years of age may have difficulty cooperating with the immunotherapy regimen and injections and, therefore, physicians need to weigh the risks and benefits of therapy in this patient population. Immunotherapy is generally not initiated in pregnant women; however, it can be safely continued in women who have been on treatment prior to becoming pregnant. Special consideration must also be given to the use of immunotherapy in the elderly since these patients often have comorbid medical conditions that may increase the risk of experiencing immunotherapy-associated adverse events. Finally, some physicians feel uncomfortable about manipulating the immune system in patients with autoimmune disorders, immunodeficiency syndromes, or malignant disease. However, there is no solid evidence that allergen-specific immunotherapy is actually harmful to these patients, provided the risks and benefits of therapy in these patients have been considered [5].

## Efficacy

### Venom immunotherapy

Venom immunotherapy provides rapid protection against Hymenoptera stings. There is a residual risk of systemic reactions of approximately 5-10% after completion of venom immunotherapy; however, when reactions to stings do occur following therapy, they are typically mild [5]. Clinical features such as a notable history of very severe reactions to a sting, systemic reactions during immunotherapy, and treatment duration of less than 5 years have been associated with a greater likelihood of relapse following the discontinuation of venom immunotherapy [7].

### Allergic rhinitis

Allergen immunotherapy is an effective treatment for allergic rhinitis, particularly for patients with intermittent (seasonal) allergic rhinitis caused by pollens [5,8] It has also been shown to be effective for the treatment of allergic rhinitis caused by tree pollen, grass pollen and ragweed pollen, house dust mites, cat and dog dander, alternaria, and cockroach. Often patient’s symptoms improve even when they were resistant to conventional drug therapy [5,9].

Evidence suggests that at least 3 years of allergen-specific immunotherapy provides beneficial effects in patients with allergic rhinitis that can persist for several years after discontinuation of therapy [10,11]. In Canada, most allergists consider stopping immunotherapy after 5 years of adequate treatment. Immunotherapy may also reduce the risk for the future development of asthma in patients with allergic rhinitis [5].

### Asthma

Immunotherapy has been shown to be effective against allergic asthma caused by grass, ragweed, house dust mites, cat and alternaria [6,12]. A Cochrane review of 75 randomized controlled trials examining the use of allergen-specific immunotherapy in asthma management confirmed its efficacy in reducing asthma symptom scores and medication requirements, and improving airway hyperresponsiveness [1]. Similar benefits have been noted with sublingual immunotherapy [13], which is expected to be approved in Canada in the near future. Evidence also suggests that allergen-specific immunotherapy may prevent the onset of asthma in atopic individuals [14,15]. One study in children with grass and/or birch pollen allergy found that only 26% of subjects treated with immunotherapy developed asthma 3 years after completion of treatment compared to 45% who were not treated with immunotherapy [15]. Allergen-specific immunotherapy may also modify the progression of established asthma in children. A study published in the 1960s found that 70% of treated children no longer had asthma 4 years after completing immunotherapy compared to 19% of untreated control subjects, and these results were sustained up to 16 years of age [16]. However, there is no current evidence that immunotherapy influences the evolution of established asthma in adults.

## Patient selection

The decision to proceed with allergen-specific immunotherapy should be made on a case-by-case basis, taking into account individual patient factors such as the degree to which symptoms can be reduced by avoidance measures and pharmacological therapy, the amount and type of medication required to control symptoms, and the adverse effects of pharmacological treatment [7].

Furthermore, patients selected for immunotherapy should be cooperative and compliant. Patients who have a history of noncompliance or who are mentally or physically unable to communicate clearly with the treating physician may be poor candidates for immunotherapy. Inability to communicate clearly with the physician will make it difficult for the patient to report signs and symptoms suggestive of systemic reactions [7].

### Venom hypersensitivity

Before deciding to proceed with venom immunotherapy, it is important to consider the natural history of venom allergy. Patients who have experienced systemic symptoms after a sting are at much greater risk of severe systemic reactions on subsequent stings compared with patients who have had only local reactions. The frequency of systemic reactions to stings ranges between 5-10% in those who have a history of large local reactions compared to 30-70% in those who have had a previous systemic reaction. In general, children are at lower risk of repeated systemic reactions, as are those with a history of milder reactions [5].

It is also important to consider occupational and geographic factors that may increase the likelihood of future stings. For example, bee stings are much more common in beekeepers, their families, and their neighbors. Yellow-jacket stings are more common in certain occupations such as bakers, grocers and outdoor workers [5].

### Allergic rhinitis

**Figure 1:**
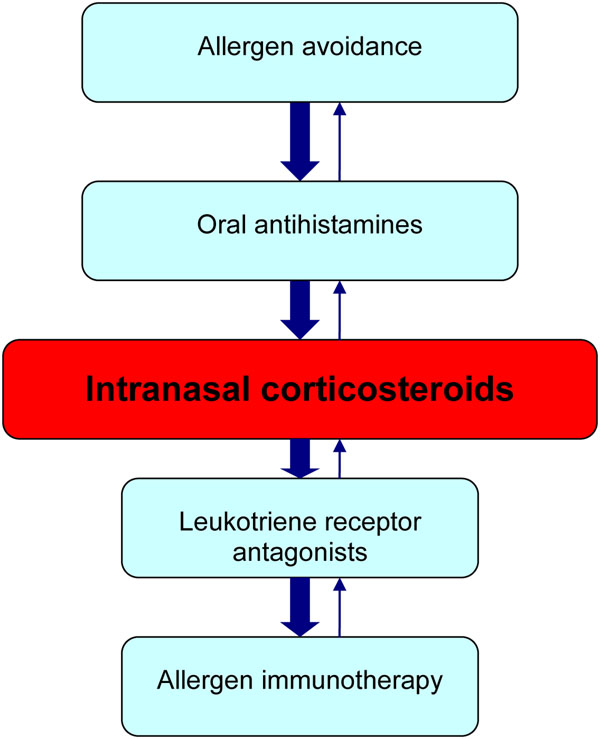
**A simplified, stepwise algorithm for the treatment of allergic rhinitis. Note: Treatments can be used individually or in any combination.**

Patients with allergic rhinitis who are unable to sleep because of symptoms or whose symptoms interfere with their work or school performance are particularly good candidates for immunotherapy. Those that experience adverse side effects from pharmacological therapy, such as nosebleeds from intranasal steroids or excessive drowsiness from antihistamines, and those who find pharmacotherapy inconvenient or ineffective, may also be appropriate candidates for immunotherapy [5]. A flow diagram for the management of allergic rhinitis is provided in Figure 1 (for more information on the management of allergic rhinitis, please see the Allergic Rhinitis article in this supplement).

### Asthma

**Figure 2:**
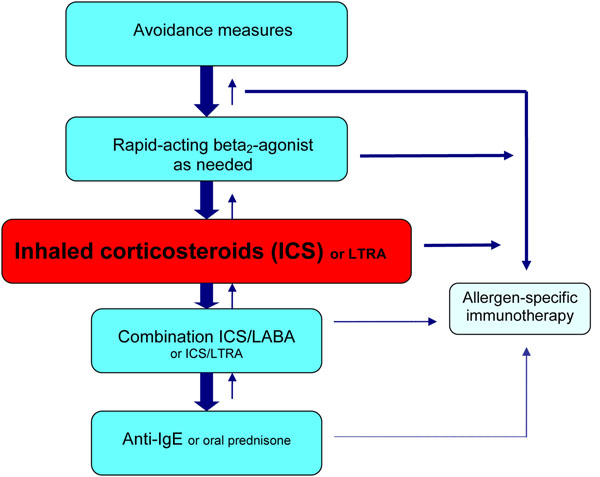
**A simplified, stepwise algorithm for the treatment of asthma.*** ICS: inhaled corticosteroid; LTRA: leukotriene receptor antagonist; LABA: long-acting beta_2_-agonist; IgE: immunoglobulin E*** Note: Treatments can be used individually or in any combination.**

As with allergic rhinitis and venom allergy, the use of allergen-specific immunotherapy in asthma should be considered on a case-by-case basis. It can be used prior to a trial of inhaled corticosteroid (ICS) therapy in patients with very mild allergic asthma and concomitant allergic rhinitis and as add-on therapy in patients using ICSs alone [12]. Allergen-specific immunotherapy may also be considered in patients using combination inhalers, ICS/leukotriene receptor antagonists (LTRAs) and/or omalizumab if asthma symptoms are controlled. (see Figure 2; for more information on the management of asthma, please see the Asthma article in this supplement). However, to reduce the risk of serious reactions, asthma symptoms must be controlled and the forced expiratory volume in 1 second (FEV_1_) should be greater than 70% predicted at the time immunotherapy is administered.

## Immunotherapy administration and schedules

Allergen-specific immunotherapy carries the risk of anaphylactic reactions (serious allergic reactions that are rapid in onset and may cause death) and, therefore, should only be prescribed by physicians who are adequately trained in the treatment of allergy and the use of immunotherapy (such as allergists and immunologists). The injections must be given where a physician is present, and in clinics that are equipped to manage possible life-threatening reactions.

Before immunotherapy is started, patients should understand its benefits, risks, and costs. Counseling should also include the expected onset of efficacy and duration of treatment, as well as the risk of anaphylaxis and importance of adhering to the immunotherapy schedule. An assessment of the patient’s current health status should be made before the administration of immunotherapy injections to determine whether there have been any recent changes in the patient’s health that may require modifying or withholding treatment (e.g., uncontrolled/symptomatic asthma or exacerbation of allergy symptoms) [7].

Once it has been determined that the patient is suitable for immunotherapy, the allergist/immunologist will use extracts of clinically relevant allergens for immunotherapy treatment sets. Allergen extracts are commercially available for most of the commonly recognized allergens (e.g., grass and tree pollen, house dust mites, insect venom). When possible, standardized extracts should be utilized to prepare treatment sets since the efficacy and safety of immunotherapy are dependent on the quality of the allergen extracts used. In choosing the components for a clinically relevant allergen immunotherapy extract, the physician must also be familiar with local and regional aerobiology and indoor and outdoor allergens, paying special attention to potential allergens in the patient’s own environment. Table 2 provides the timing and concentration of common pollen and mould allergens across the various geographic regions in Canada [6,7].

**Table 2:** Timing and concentration of suspect pollens and mould spores in various geographic areas across Canada [6]

	Tree pollen	Grass pollen	Weed pollen	Mould spores
**British Columbia (Coastal)**	• Season: early February to mid-July • Primarily deciduous trees (alder, birch, poplar, elm, oak)	• Season: end of April to September • Highest grass concentrations: early June to mid-July	• Not usually a major factor; no native ragweed	• Levels higher in the spring; increase further in September and October • Most prevalent spores: Cladosporium and basidiomycetes

**British Columbia (Interior)**	• Season: late March to mid-July • Primarily deciduous trees (willow, birch, poplar)	• Season starts in early May in southern parts of the province; starts up to 1 month later in northern parts	• Ragweed is minimal	• Cladosporium can occur from April to late fall

**Prairies**	• Season starts in the first week of April and continues through June&#8226 Main deciduous trees: birch and poplar; alder, maple, elm, oak, ash, and willow may also contribute	• Season starts in mid‐May and continues to the end of September&#8226 Peak season is usually mid‐June to early July	• Most common weeds: nettles or sage brush&#8226 Some ragweed, especially in Manitoba)	• Can occur through the spring, summer, and early fall&#8226 Alternaria and Cladosporium are the predominant moulds

**Ontario and Quebec**	• Season starts early April in southern Ontario and Quebec; may occur 6 weeks later in northern areas • In southern Ontario, most common are deciduous trees (birch, poplar, oak, maple, ash, elm, mulberry, willow, chestnut, hickory) • In northern Ontario, birch and poplar most common • In Quebec, ash, poplar, birch most common; maple, alder and oak are less prevalent	• Season starts mid-to-late May; a couple of weeks later in northern areas • Latter part of May and mid-June are peak seasons for grass pollination	• Ragweed season in Southern Ontario and Southwestern Quebec begins early-to-mid August • Reaches peak in late August/early September • Stops at first frost • Nettle and plantain can also contribute	• Occur during spring, summer and fall months • Concentrations may be higher late summer to fall months in Quebec • Alternaria and Cladosporium are the predominant moulds

**Maritimes**	• Season in New Brunswick and Nova Scotia: late March to last week of June • Primarily deciduous trees (birch, poplar, alder, maple, oak, and ash)	• Season: mid-May to end of September • Peaks in early June	• Ragweed: early August to end of September	• Levels higher during the late summer and early fall months • Alternaria and Cladosporium are the predominant moulds

Typically, allergen-specific immunotherapy consists of two phases: a build-up phase (also known as up-dosing or induction) and a maintenance phase. During the build-up phase, the patient receives weekly injections, starting with a very low dose, with gradual increases in dose over the course of 5-8 months. After this period, the patient has usually built up sufficient tolerance to the allergen such that a maintenance (therapeutic) dose has been reached. During the maintenance phase, the patient receives injections of the maintenance dose every 3 to 4 weeks, usually for a period of 3 to 5 years. After this period, many patients experience a prolonged, protective effect and, therefore, consideration can be given to stopping therapy [7].

Accelerated schedules, such as rush or cluster immunotherapy, may also be used and involve the administration of several injections at increasing doses on a single visit. With cluster immunotherapy, two or three injections (at increasing doses) are given sequentially in a single day of treatment on non-consecutive days. Rush immunotherapy entails administering incremental doses of the allergen at intervals varying between 15 and 60 minutes over 1 to 3 days until the target maintenance dose is achieved. Although accelerated schedules offer the advantage of achieving the therapeutic dose much earlier than conventional immunotherapy schedules, they may also be associated with an increased risk of systemic reactions, and are not typically used in Canada for respiratory allergies [5,7].

Pre-seasonal immunotherapy preparations that are administered on an annual basis are also available. They offer a much shorter build-up phase. Sublingual preparations are also expected to be approved in Canada in the near future. These should provide patients with effective therapeutic options. Although patients will be able to self-administer the sublingual formulations, close monitoring by a physician will still be required.

Patients receiving maintenance immunotherapy should be followed regularly to: assess the efficacy of treatment; monitor adverse reactions; assess patient compliance with therapy; and determine whether immunotherapy can be discontinued or if dose/schedule adjustments are required. For example, dose reductions may need to be considered during periods when the patient is exposed to increased allergen levels or when he/she is experiencing an exacerbation of symptoms.

At present, there are no specific tests or clinical markers that will distinguish between patients who will relapse and those who will remain in long-term clinical remission after discontinuing allergen immunotherapy. Therefore, the decision to continue immunotherapy beyond 3 to 5 years should be based on individual patient factors such at the severity of the disease, benefits sustained from treatment, reaction history, patient preference, and treatment convenience [7].

## Safety

Allergen-specific immunotherapy is generally safe and well-tolerated when used in appropriately selected patients. However, local and systemic reactions may occur. Local reactions, such as redness or itching at the injection site, can generally be managed with local treatment (e.g., cool compresses or topical corticosteroids) or oral antihistamines. Systemic reactions occur in approximately 1-4% of patients on allergen immunotherapy [6] and can be mild to severe. The most severe reaction is anaphylaxis. Fatal anaphylactic reactions are rare, occurring in an estimated 1 in every 8 million doses of immunotherapy administered [6].

There are numerous signs and symptoms of anaphylaxis that involve the skin, gastrointestinal and respiratory tracts, and cardiovascular system (see Table 3) [17]. These symptoms typically develop within 30 minutes after the immunotherapy injection. In fact, most documented fatalities (73%) have occurred within 30 minutes of the injection [6]. It is important to note that the signs and symptoms of anaphylaxis are unpredictable and may vary from patient to patient. Therefore, the absence of one or more of the common symptoms listed in Table 3 does not rule out anaphylaxis, and should not delay immediate treatment [6].

**Table 3:** Signs and symptoms of anaphylaxis [17]

Signs/symptoms	Incidence
Urticaria, angioedema	87%
Dyspnea	59%
Dizziness, syncope	33%
Diarrhea, abdominal cramps	29%
Flushing	25%
Upper airway edema	21%
Nausea, vomiting	20%
Hypotension	15%
Rhinitis	8%
Itch without rash	5%
Seizure	1%

In the event of anaphylaxis, the treatment of choice is epinephrine administered by intramuscular injection into the lateral thigh (see Anaphylaxis article in this supplement for more information on the diagnosis and management of anaphylaxis). Adjunctive therapies such as antihistamines, bronchodilators and systemic corticosteroids may also be used, but should never be given prior to or replace epinephrine in the treatment of anaphylaxis. In severe cases, intravenous saline or supplemental oxygen may be required [5-7].

Following a systemic reaction to immunotherapy, consideration should be given to reducing the therapeutic dose or to possibly discontinuing therapy, particularly if the patient has repeated systemic reactions following injections [5-7].

## Conclusions

Allergen-specific immunotherapy is a potentially disease-modifying therapy that is effective for the treatment of allergic rhinitis/conjunctivitis, allergic asthma and stinging insect hypersensitivity. Although it is still unclear exactly how this form of therapy works, immunotherapy has been associated with a shift from Th2 to Th1 immune responses, and the production of T regulatory cells that dampen the immune response to relevant allergens. When used in appropriately-selected patients, allergen-specific immunotherapy is extremely safe. This form of therapy, however, does carry the risk of anaphylactic reactions and, therefore, should only be prescribed by physicians who are adequately trained in the treatment of allergy. Furthermore, immunotherapy should be administered only by physicians who are equipped to manage life-threatening anaphylaxis.

## Key take-home messages

• Allergen-specific immunotherapy is a potentially disease-modifying therapy that is effective for the treatment of allergic rhinitis/conjunctivitis, allergic asthma and stinging insect hypersensitivity.

• Allergen immunotherapy is contraindicated in patients on beta-blockers, those with uncontrolled or severe asthma, or those with significant co-morbid cardiovascular disease.

• The decision to proceed with allergen immunotherapy should be made on a case-by-case basis, taking into account individual patient factors such as disease severity, efficacy of avoidance measures and pharmacological therapy, and patient preferences.

• Allergen immunotherapy carries the risk of anaphylactic reactions and, therefore, should only be prescribed by physicians who are adequately trained in the treatment of allergy

• Injections must be given under medical supervision in clinics that are equipped to manage life-threatening anaphylaxis.

## Competing interests

Dr. William Moote has received consulting fees or honoraria for continuing education from AstraZeneca, Merck and Amgen.

Dr. Harold Kim is the past president of the Canadian Network for Respiratory Care and co-chief editor of *Allergy*, *Asthma and Clinical Immunology*. He has received consulting fees and honoraria for continuing education from AstraZeneca, GlaxoSmithKline, Graceway Pharmaceuticals, King Pharma, Merck Frosst, Novartis, and Nycomed.
